# Comparative study of fractional flow reserve and diastolic pressure ratio using a guidewire with a sensor for measuring intravascular pressure

**DOI:** 10.1097/MD.0000000000032578

**Published:** 2022-12-30

**Authors:** Hiroki Kojima, Hideki Ishii, Akihito Tanaka, Hiroshi Funakubo, Toshiaki Kato, Yusaku Shimbo, Toshiki Kawamiya, Yachiyo Kuwatsuka, Masahiko Ando, Toyoaki Murohara

**Affiliations:** a Department of Cardiology, Nagoya University Graduate School of Medicine, Nagoya, Japan; b Department of Cardiology, Nagoya Ekisaikai Hospital, Nagoya, Japan; c Department of Cardiovascular Medicine, Gunma University Graduate School of Medicine, Maebashi, Japan; d Department of Cardiology, Kariya Toyota General Hospital, Kariya, Japan; e Department of Cardiology, Tsushima City Hospital, Tsushima, Japan; f Department of Advanced Medicine, Nagoya University Hospital, Nagoya, Japan.

**Keywords:** coronary artery, diastolic pressure ratio (dPR), fractional flow reserve (FFR)

## Abstract

This study aimed to evaluate the correlation and diagnostic agreement between diastolic pressure ratio (dPR) and fractional flow reserve (FFR) in a Japanese real-world setting.

**Design:**

Prospective multicenter observational study

**Methods:**

This study included 100 patients with intermediate coronary artery stenosis at 4 Japanese hospitals. For these lesions, FFR and dPR were measured using a guidewire with a sensor and a monitor to measure intravascular pressure. The correlation and diagnostic agreement between FFR and dPR were assessed. When both FFR and dPR were negative or positive, the results were considered to be concordant. When one was positive and the other was negative, the result was regarded as discordant (positive discordance, FFR > 0.80 and dPR ≤ 0.89; negative discordance, FFR ≤ 0.80 and dPR > 0.89).

**Results:**

Overall, the FFR and dPR were well-correlated (*R* = 0.841). FFR and dPR were concordant in 89% of cases (concordant normal, 43%; concordant abnormal, 46%) and discordant in 11% (positive discordance, 7%; negative discordance, 4%). No significant difference was observed in the rate of concordant results between patients with and without diabetes mellitus. The diagnostic concordance rate was significantly different among the 3 coronary arteries (right coronary artery, 93.3%; left anterior descending artery, 93.2%; and left circumflex artery, 58.3%; *P* = .001). Additionally, the rate of concordant results tended to be higher when using intravenous administration of adenosine than when using intracoronary bolus injection of nicorandil (adenosine, 95.1%; nicorandil, 84.7%; *P* = .103).

**Conclusion:**

We found that dPR was highly correlated with FFR, and diagnostic discordance was observed in 11% of the lesions. Several factors, including lesion location and medication for hyperemia, may cause the diagnostic discordance between dPR and FFR.

## 1. Introduction

Fractional flow reserve (FFR) is the most established invasive physiological test for assessing the functional severity of coronary artery stenosis.^[1–3]^ Recently, instantaneous wave-free ratio (iFR) was introduced as a useful alternative, and has been shown to be clinically noninferior to FFR.^[4,5]^ Other resting pressure-derived indices have been introduced and expanded to clinical practice.^[6–10]^ The diastolic pressure ratio (dPR), which identifies the average ratio of resting distal coronary pressure (Pd) to aortic pressure (Pa) over the entire diastole, is becoming a popular resting index.^[6]^ The diagnostic performance of iFR and other new resting indices, including dPR, can be equally utilized in the prediction of myocardial ischemia.^[6,7]^ However, some differences among the various resting physiological indexes may exist,^[9]^ and data regarding the direct comparison of other resting indexes with FFR are still limited.^[7–9]^ Furthermore, some patient- and lesion-related factors have been reported to influence the discrepancy between FFR and iFR; however, these issues have not been fully validated in dPR and others indices.^[11]^ The aim of the present study was to assess the correlation and diagnostic agreement between FFR and dPR in a real-world setting.

## 2. Methods

### 2.1. Study population

This prospective study was a single-arm, nonrandomized, noncontrolled multicenter investigator-initiated clinical trial (jRCTs042200015) sponsored by Zeon Medical, Inc. Between June 2020 and June 2022, we prospectively recruited adult patients aged ≥ 20 years who underwent coronary catheterization for suspected coronary artery disease at 4 centers. The indication for invasive coronary angiography was based on the guideline on diagnosis of chronic coronary heart diseases.^[12]^ Inclusion criteria after diagnostic coronary angiography were: the existence of angiographically intermediate stenosis (50–75%, and lesion length < 40 mm) by visual assessment in a major epicardial coronary artery and requirement of functional assessment judged by the attending physician. The narrowest lesion was analyzed as the subject of this study when a patient had ≥ 2 lesions that fulfilled the criteria. The exclusion criteria were as follows: if the device could not be delivered 3.5 cm beyond the target lesion, a prior history of stenting, coronary bypass grafting or myocardial infarction in the target vessel, second- or third- degree atrioventricular block, New York Heart Association classes III or IV, possibility of pregnancy, severe peripheral artery disease, contraindication to antiplatelet therapy and anticoagulant, allergy to contrast media or medication for hyperemia (adenosine or nicorandil), severe left ventricular dysfunction defined as an left ventricular ejection fraction <35%, and active gastrointestinal bleeding. If patients met these criteria, they were deemed inappropriate to participate in the study by the attending physician. The study protocol was approved by the institutional ethics committee and written informed consent was obtained from all patients before the procedure.

### 2.2. Coronary angiography and physiological assessment

Diagnostic coronary angiography was performed by using 5- or 6-Fr catheters. Physiological assessment was then performed according to standard technique.^[13,14]^ A pressure guidewire with optical fibers (OptWire® II or III, Zeon Medical Inc., Tokyo, Japan) was used for intracoronary pressure measurements. The guidewire was calibrated outside the body and advanced to the catheter tip. After equalization of pressure between the guidewire and the tip of the catheter was confirmed, the wire was advanced and placed > 3–3.5 cm distal to the stenosis. First, the coronary pressure (Pd) and aortic pressure (Pa) were measured at this position and dPR, which is the ratio of resting Pd to Pa during diastole, was recorded. Subsequently, hyperemia was induced by the intravenous administration of adenosine (140–180 μg/kg/min) or intracoronary bolus injection of nicorandil (2 mg). During hyperemia, Pd and Pa were measured, and the FFR, which is the ratio of average Pd to Pa during hyperemia, was recorded. After the measurement, the guidewire was gradually pulled back, and retracted to the catheter tip to confirm the absence of any significant pressure drift, defined as > 0.03 or < −0.03. The thresholds for a significant stenosis of FFR and dPR were defined to be ≤ 0.80 and ≤ 0.89, respectively. When both FFR and dPR were negative (FFR > 0.80 and dPR > 0.89) or positive (FFR ≤ 0.80 and dPR ≤ 0.89), they were regarded as concordant. When one was positive and the other was negative (FFR > 0.80 and dPR ≤ 0.89, or FFR ≤ 0.80 and dPR > 0.89), the result was regarded as discordant. In cases of discordance, a FFR > 0.80 and dPR ≤ 0.89 was defined as positive discordance, and FFR ≤ 0.80 and dPR > 0.89 as negative discordance.^[11]^

### 2.3. Statistical analysis

Categorical variables were expressed as numbers and percentages. Continuous variables are expressed as mean ± standard deviation or median (interquartile range [IQR]). Correlations between FFR and dPR were assessed using Pearson correlation coefficients. Categorical data were compared using the chi-squared or Fisher exact test as appropriate. Continuous variables were compared using Student unpaired *t*-test or Wilcoxon rank-sum test as appropriate. To assess the influence of potential patient/lesion factors, we compared the results among the groups stratified according to the presence of diabetes mellitus (DM), target vessels, and the use of nicorandil or adenosine. Statistical significance was set at *P* < .05. Statistical analyses were performed using STATA version 15 (StataCop, College Station, TX).

## 3. Results

The baseline clinical characteristics of the patients are described in Table 1. In a total of 100 patients, the mean age was 70 ± 10 years, 73 (73%) were male, and 50 (50%) had DM. When comparing patients with and without DM, those with DM were significantly younger and more frequently had dyslipidemia and silent ischemia. Table 2 lists the procedural characteristics and measurement results. In most patients, the target vessel was the left anterior descending artery (73%). As a drug to obtain hyperemia, adenosine was used in 41% of patients, and nicorandil in 59%. There was no harmful event in the study.

**Table 1:** Baseline clinical characteristics in overall patients, and those with and without diabetes mellitus.

	Overall	DM−	DM +	*P* value
	N = 100	N = 50	N = 50	
Age ± SD, yr	70 ± 10	72 ± 9	68 ± 11	.047
Male, n (%)	73 (73%)	37 (74%)	36 (72%)	.822
Height ± SD, cm	161 ± 8	160 ± 8	162 ± 8	.280
Body weight ± SD, kg	64 ± 13	62 ± 12	67 ± 15	.053
Hypertension, n (%)	74 (74%)	34 (68%)	40 (80%)	.171
Dyslipidemia, n (%)	70 (70%)	30 (60%)	40 (80%)	.029
Smoking, n %)				.976
None	39 (39%)	20 (40%)	19 (38%)	
Past	45 (45%)	22 (44%)	23 (46%)	
Current	16 (16%)	8 (16%)	8 (16%)	
History of myocardial infarction, n (%)	15 (15%)	8 (16%)	7 (14%)	.779
LVEF ± SD, %	62 ± 9	63 ± 9	61 ± 9	.331
				
Clinical presentation, n (%)				.015
Stable angina	68 (68%)	40 (80%)	28 (56%)	
Old myocardial infarction	4 (4%)	0 (0%)	4 (8%)	
Silent ischemia	28 (28%)	10 (20%)	18 (36%)	
*Laboratory test*				
eGFR ± SD, mL/min/1.73m²	59.4 ± 24.7	64.0 ± 18.8	54.7 ± 28.9	.058
Glucose ± SD, mg/dL	135 ± 45	115 ± 25	154 ± 53	<.001
HbA1c ± SD, %	6.5 ± 1.1	6.0 ± 0.4	7.0 ± 1.3	<.001
Total cholesterol ± SD, mg/dL	177 ± 37	185 ± 41	170 ± 32	.045
HDL-cholesterol ± SD, mg/dL	52 ± 15	53 ± 15	50 ± 14	.228
LDL-cholesterol ± SD, mg/dL	97 ± 30	105 ± 32	89 ± 25	.006
Triglyceride ± SD, mg/dL	154 ± 85	149 ± 87	159 ± 83	.555
Hemoglobin ± SD, g/dL	13.4 ± 1.7	13.6 ± 1.3	13.1 ± 1.9	.155
Hematocrit ± SD, %	39.9 ± 4.7	40.4 ± 3.7	39.3 ± 5.5	.258
CRP, median (interquartile range), mg/dL	0.1 (0.0–0.2)	0.1 (0.0–0.2)	0.1 (0.1–0.2)	.552

DM = diabetes mellitus; DM− = patients without DM; DM+ = patients with DM, eGFR = estimated glomerular filtration rate; HbA1c, glycated hemoglobin A1c, HDL = high-density lipoprotein, LDL = low-density lipoprotein, LVEF = left ventricular ejection fraction, SD = standard deviation.

**Table 2:** Procedural characteristics and measurement results in overall patients and those with and without diabetes mellitus.

	Overall	DM−	DM +	*P* value
	N = 100	N = 50	N = 50	
Systolic blood pressure ± SD, mm Hg	137 ± 23	137 ± 25	137 ± 22	.936
Diastolic blood pressure ± SD, mm Hg	74 ± 13	74 ± 12	75 ± 14	.690
Heart rate ± SD, bpm	70 ± 12	68 ± 12	72 ± 12	.115
Target vessel, n (%)				.623
Right coronary artery	15 (15%)	6 (12%)	9 (18%)	
Left anterior descending artery	73 (73%)	37 (74%)	36 (72%)	
Left circumflex artery	12 (12%)	7 (14%)	5 (10%)	
Drug, n (%)				.839
Intravenous adenosine	41 (41%)	20 (40%)	21 (42%)	
Intracoronary nicorandil	59 (59%)	30 (60%)	29 (58%)	
Measurement results				
FFR ± SD	0.78 ± 0.11	0.78 ± 0.10	0.78 ± 0.11	.865
dPR ± SD	0.86 ± 0.11	0.86 ± 0.11	0.85 ± 0.12	.493

dPR = diastolic pressure ratio, FFR = fractional flow reserve.

**Figure 1.**
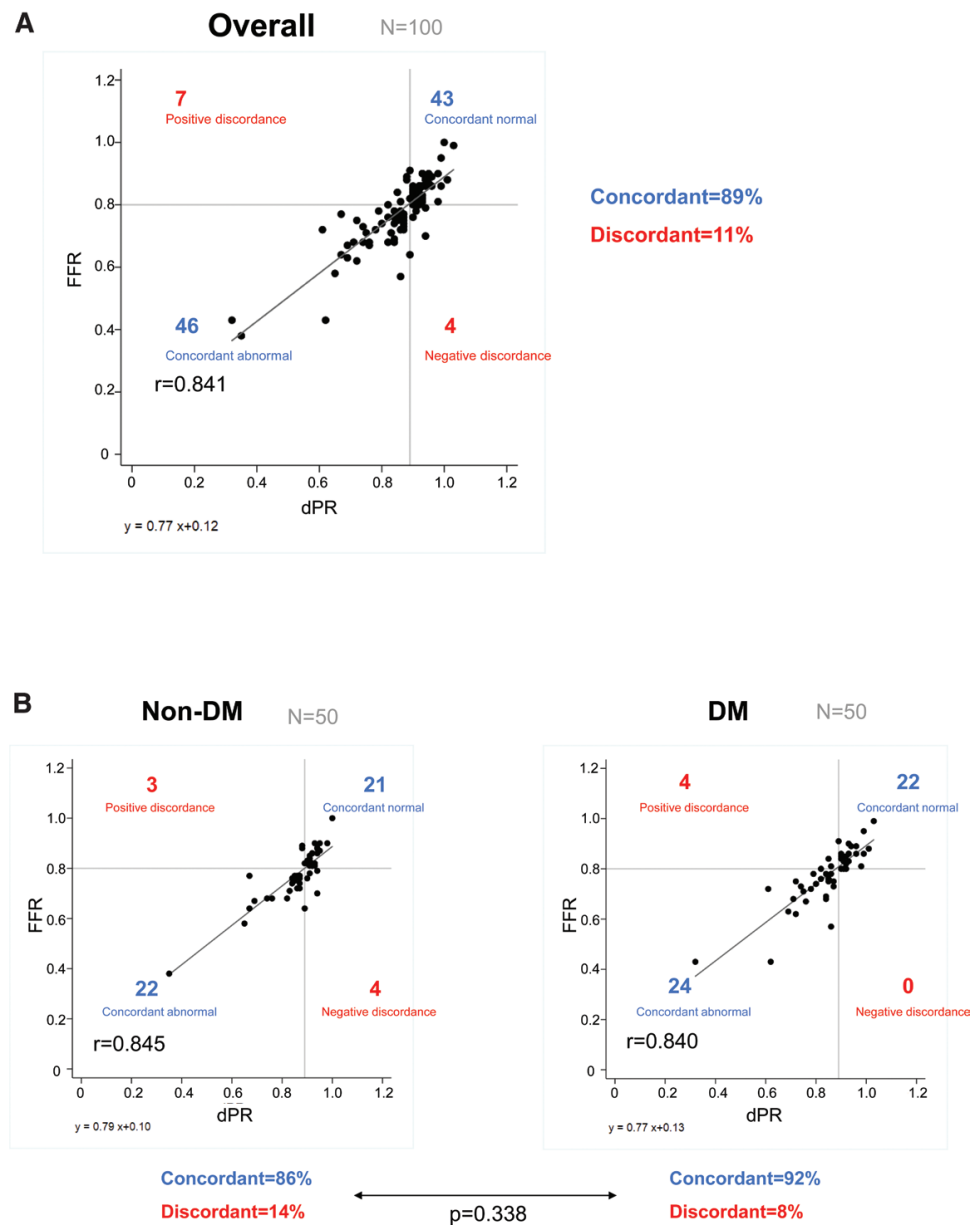
Correlation between diastolic pressure ratio and fractional flow reserve in all patients (A), the patients without diabetes mellitus (B), and those with diabetes mellitus (C).

The mean FFR and dPR were 0.78 ± 0.11, and 0.86 ± 0.11, respectively. Figure 1A shows the correlation between FFR and dPR in all patients. Overall, the FFR and dPR were well-correlated (*R* = 0.841). Furthermore, FFR and dPR were diagnostically concordant in 89% (concordant normal, 43%; concordant abnormal, 46%), and discordant in 11% (positive discordance: high FFR and low dPR, 7%; negative discordance: low FFR and high dPR, 4%).

Figure 1B shows the correlation between FFR and dPR in patients with DM (n = 50) and in those without DM. There was no significant difference in the rate of concordant results between patients with and without DM (DM, 92%; non-DM, 86%, *P* = .338). In patients without DM, 3 discordant results were positive discordance and 4 negative discordance. In patients with DM, all 4 discordant results were positive discordant.

**Figure 2.**
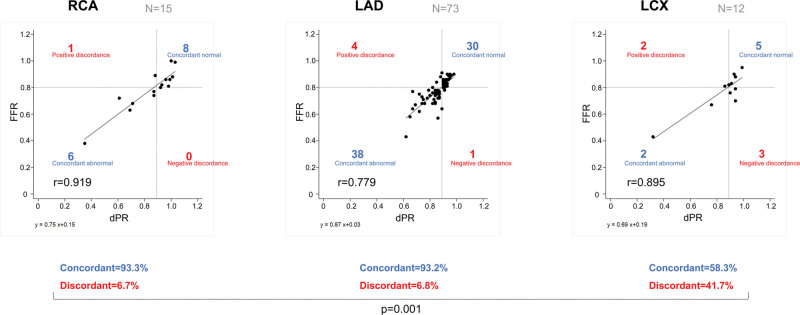
Correlation between diastolic pressure ratio and fractional flow reserve in right coronary artery (A), left anterior descending artery (B), and left circumflex artery (C).

Figure 2 shows the correlation between the FFR and dPR in each coronary vessel (right coronary artery, n = 15; left anterior descending artery, n = 73; and left circumflex artery, n = 12). The concordance rate was significantly different among the 3 coronary arteries (right coronary artery, 93.3%; left anterior descending artery, 93.2%; and left circumflex artery, 58.3%; *P* = .001). In 12 patients with left circumflex artery assessment, FFR and dPR were discordant in 5 patients—two had positive discordance (high FFR and low dPR) and 3 had negative discordance (low FFR and high dPR).

**Figure 3.**
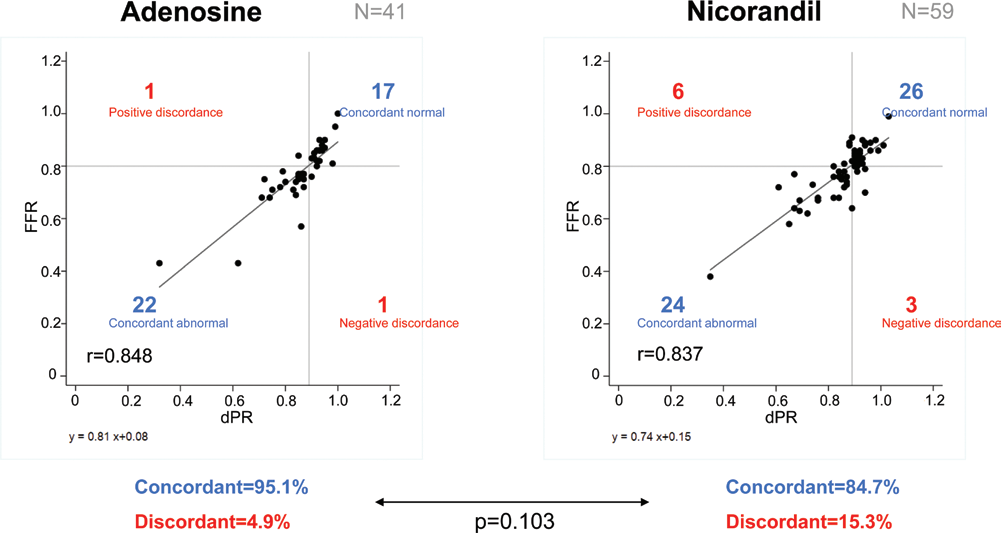
Correlation between diastolic pressure ratio and fractional flow reserve when using intravenous administration of adenosine (A), and intracoronary bolus injection of nicorandil (B).

Figure 3 shows the correlation between FFR and dPR when intravenous administration of adenosine or intracoronary bolus injection of nicorandil were used. No significant difference was observed, but the rate of concordant results tended to be higher when using intravenous administration of adenosine than when using intracoronary bolus injection of nicorandil (adenosine, 95.1%; nicorandil, 84.7%; *P* = .103).

## 4. Discussion

In this study, we assessed the correlation and diagnostic agreement between the FFR and dPR in a real-world Japanese setting. The main findings of the present study were as follows: overall, dPR was well correlated with FFR, and the diagnostic agreement was 89%; no significant differences were observed in the rates of concordant results of FFR and dPR between patients with and without DM; the rate of concordant results of FFR and dPR was significantly lower in the left circumflex artery; and the rates of concordant result of FFR and dPR tended to be higher when adenosine was used than when nicorandil was used.

Prior studies have reported that the diagnostic agreement between FFR and iFR is approximately 80%, and discordance exists in approximately 10–20% of cases.^[11,15–17]^ Although dPR may be considered identical to iFR with respect to diagnostic agreement with FFR,^[6,7,10]^ data regarding the direct comparison between FFR and dPR are still limited. A previous study reported that FFR and dPR were discordant in 12.5% of cases (positive discordance, 3.3%; negative discordance 9.2%).^[8]^ Similar to their results, in the present study, we reported 11% discordance (positive discordance, 7%; negative discordance, 4%) in a real-world setting. Many potential factors that may cause discordance between FFR and resting indices have been reported,^[11]^ and the overall distribution in 4 categories (concordant normal, concordant abnormal, positive discordant, and negative discordant) could differ depending on the degree of coronary artery stenosis severity across the study population.^[11,18,19]^ In this study, the rate of concordant abnormalities was relatively high, suggesting more severe coronary artery stenosis in the study population. Despite the differences from prior reports, our study demonstrated good agreement between FFR and dPR, which is consistent with prior studies.

The presence of DM is a well-known significant factor causing FFR and iFR discordance, particularly positive discordance.^[8,11,18]^ DM is associated with microvascular dysfunction, and a higher microvascular resistance can lead to lower coronary flow during hyperemia.^[11,18,20]^ Therefore, positive discordance (ex. high FFR and low iFR) may occur. In the present study, no significant difference was observed in the FFR and dPR discordance rates between patients with and without DM, although all discordant cases were categorized as positive discordance in patients with DM. This might be due to the small sample size of the study; however, the clinical impact of DM might be minimal when using FFR or dPR, as a previous study showed comparable clinical outcomes between iFR-guided and FFR-guided measurements in DM patients.^[21]^

The effect of the location of the coronary artery stenosis has also been discussed in previous studies. Many studies have suggested that stenosis of the left anterior descending artery could cause greater discordance between FFR and resting indexes.^[11,19,22]^ However, some reports showed more negative discordance in the left anterior descending artery, whereas others showed more positive discordance.^[11]^ Furthermore, another report showed that the left anterior descending artery was a predictor of positive discordance, whereas the nonleft anterior descending artery was a predictor of negative discordance.^[22]^ Therefore, this interpretation cannot be considered consistent. In our study, lesions in the left circumflex artery showed a higher discordance rate than those in the left anterior descending and right coronary arteries. However, the majority of target lesions were left anterior descending artery (79%) and a small number of lesions in the left circumflex artery and right coronary arteries; therefore, our results regarding the target vessel are inconclusive. The sizes of the domination territories vary, even in the same vessel; not only the target vessel but also the location of the lesion, is important when interpreting the discordance between resting indexes and FFR.^[11,22]^

Intravenous administration of adenosine and intracoronary bolus injection of nicorandil are widely used to induce hyperemia.^[11,22]^ To date, there is little data regarding the differences in the effects of nicorandil and adenosine on the occurrence of FFR and resting index discordance. A prior study reported that intracoronary bolus injection of nicorandil could produce more pronounced hyperemia and is the preferred method for intravenous administration of adenosine.^[23]^ Moreover, another study indicated that the duration of hyperemia varied when nicorandil was used, which might require careful interpretation.^[24]^ In the present study, no significant difference was observed; however, the rate of concordant results between FFR and dPR tended to be higher with using adenosine than with using nicorandil. Furthermore, FFR is a diagnostic reference standard, and the difference in diagnostic accuracy between adenosine and nicorandil may affect the occurrence of FFR and dPR discordance. With regard to this issue, more data are required.

## 5. Limitations

This study had several limitations. First, the sample size was relatively small. This is a serious consideration when considering some influencing factors and comparing groups. Second, hyperemia was achieved via intravenous administration of adenosine or intracoronary bolus injection of nicorandil when measuring the FFR. Additionally, FFR was used as a standard reference for diagnosis, and its accuracy could have affected the results.

## 6. Conclusions

In conclusion, dPR was highly correlated with FFR, and diagnostic discordance was observed in 11% of the patients. Our results might support the wide availability of dPR in real-world settings; however, we need to consider that several factors may cause diagnostic disagreement between FFR and dPR, including the evaluated lesion location and medication for hyperemia.

## Acknowledgments

The authors would like to thank Ms. Fumiko Sugiura, data manager of the Department of Advanced Medicine, Nagoya University Hospital.

## Author contributions

**Conceptualization:** Hiroki Kojima, Yusaku Shimbo, Toyoaki Murohara.

**Data curation:** Hiroki Kojima, Akihito Tanaka, Toshiaki Kato, Toshiki Kawamiya.

**Formal analysis:** Yachiyo Kuwatsuka, Masahiko Ando, Hideki Ishii.

**Funding acquisition:** Hideki Ishii, Masahiko Ando.

**Investigation:** Hideki Ishii, Akihito Tanaka, Hiroshi Funakubo, Toshiaki Kato, Yusaku Shimbo, Toshiki Kawamiya, Yachiyo Kuwatsuka, Toyoaki Murohara.

**Methodology:** Hiroki Kojima, Hideki Ishii, Akihito Tanaka, Hiroshi Funakubo, Toshiaki Kato, Yusaku Shimbo, Toshiki Kawamiya, Yachiyo Kuwatsuka, Masahiko Ando.

**Project administration:** Hideki Ishii.

**Resources:** Hiroshi Funakubo.

**Supervision:** Toshiaki Kato, Yusaku Shimbo, Toshiki Kawamiya, Yachiyo Kuwatsuka, Toyoaki Murohara.

**Writing—original draft:** Hiroki Kojima, Hideki Ishii.

**Writing—review and editing:** Masahiko Ando, Toyoaki Murohara, Hideki Ishii.
